# Enhancing Implantable Medical Devices: Surface Functionalization of Titanium with Quaternary Ammonium Salts for Antibacterial Adhesion Properties

**DOI:** 10.1021/acsomega.4c08503

**Published:** 2025-02-05

**Authors:** Consuelo Celesti, Daniela Iannazzo, Elpida Piperopoulos, Bartolo Gabriele, Raffaella Mancuso, Giuseppa Visalli, Alessio Facciolà, Antonio Laganà

**Affiliations:** † Department of Engineering, University of Messina, Messina, Contrada Di Dio I-98166, Italy; ‡ Laboratory of Industrial and Synthetic Organic Chemistry (LISOC), Department of Chemistry and Chemical Technologies, University of Calabria, Via Pietro Bucci 12/C, Arcavacata di Rende (CS) 87036, Italy; § Department of Biomedical and Dental Sciences and Morphofunctional Imaging, University of Messina, Messina 98125, Italy; ∥ Istituto Clinico Polispecialistico C.O.T., Cure Ortopediche Traumatologiche s.p.a., Messina 98124, Italy

## Abstract

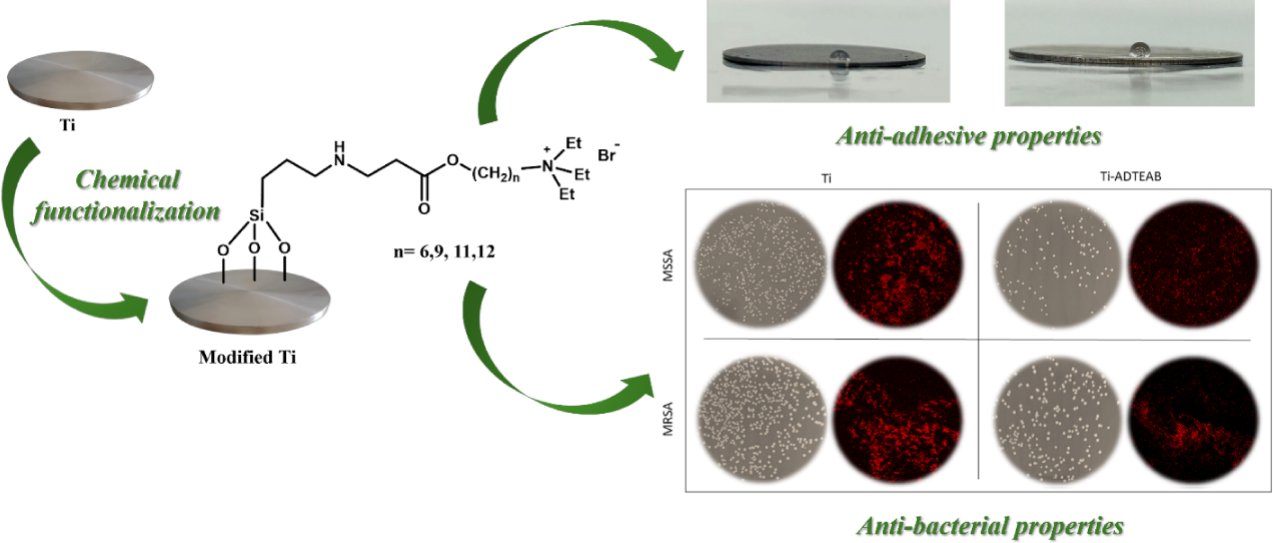

Bacterial colonization of titanium-based materials used in implantable medical devices represents a significant challenge in the dental and orthopedic fields, often leading to infections and implant failure. This study reports the surface modification of titanium discs with ammonium salts containing carbon atom chains of different lengths (from 6 to 12) to provide antibacterial properties to the modified metal surfaces while maintaining their biocompatibility. The chemically modified samples have been characterized by ATR-FTIR and SEM-EDX analyses and evaluated for roughness and hydrophilic behavior. This surface modification not only provides hydrophobic properties to titanium surfaces but also introduces a hindering environment for bacterial adhesion. Antibacterial tests performed against methicillin-sensitive and methicillin-resistant *Staphylococcus aureus* strains demonstrated a proportional increase in antibacterial activity with increasing carbon chain length. The best antibacterial performance is reported for the sample containing 12 carbon atoms (Ti-ADTEAB), which showed inhibition values of 87.5 and 86.6% for the sensitive and resistant strains, respectively. The results suggest that this surface modification could lead to a new generation of implantable medical devices with improved patient outcomes by reducing the risk of postoperative infections.

## 1 Introduction

Surface modification of implantable medical devices is a crucial step in reducing the risk of infection and improving the endurance of the device.^1^ Titanium, due to its biocompatibility and mechanical properties, is widely used in orthopedic, dental, and cardiovascular implants^2,3^ However, the colonization of bacteria on the surface of these devices can lead to serious complications such as infection and implant failure.^4^ Therefore, developing titanium surface modifications with antibacterial adhesion properties is essential in preventing biofilm formation and reducing infection rates.^5,6^ Several techniques are employed to modify titanium surfaces for antibacterial adhesion properties.^7,8^ One common method is physical surface modification, which includes roughening the surface through techniques such as sandblasting,^9^ acid etching,^10^ or laser treatment.^11^ These physical modifications increase the surface area, producing materials that decrease bacterial attachment while promoting osseointegration and cell proliferation.^12,13^ However, it has been observed that these treatments do not clearly influence time-dependent biofilm formation, and the effect of surface roughness appears to be bacterial species-dependent.^14^

Chemical surface modification is another approach to enhance the antibacterial properties of titanium surfaces.^15^ Some modification methods are based on the immobilization of antibacterial agents on the metal surface.^16^ This can be achieved using biomolecules such as antimicrobial peptides,^17^ silver nanoparticles,^18,19^ or antibiotics, which possess inherent antimicrobial properties.^20^ These agents can be covalently attached or physically adsorbed onto the titanium surface, providing a sustained release of antimicrobial activity.^21,22^ The drawbacks of these methods are mainly due to the low concentration of loaded antimicrobial agents, which can be rapidly released into human fluids with the possible onset of drug resistance phenomena and the poor efficiency for long periods.^23^ Another investigated chemical modification technique is surface coating with biocompatible materials that possess inherent antibacterial properties, such as hydroxyapatite (HA) or tricalcium phosphate (TCP).^24−26^ Even if these coatings can enhance osseointegration and prevent bacterial adhesion to a certain extent^27,28^ often fail to inhibit the growth of pathogenic bacteria.^28^ In recent years, advanced surface modification techniques, such as plasma immersion ion implantation (PIII), have gained attention.^29^ PIII allows for the incorporation of antibacterial elements, such as silver or copper ions, directly into the titanium surface.^30^ This technique provides a durable and long-lasting antibacterial effect and can prevent bacterial resistance development.^31^ However, the use of metal coatings can cause serious toxicity issues and long-term damage due to the accumulation of metals in human tissues.^32^

Based on these considerations, we have investigated the possibility of developing intrinsic antibacterial metal devices by covalently linking bioactive ammonium salts onto the surface of titanium metal substrates. Ammonium salts, particularly quaternary ammonium salts (QASs), have been extensively studied for their antimicrobial properties and ability to prevent bacterial adhesion and biofilm formation.^33^ These molecules have a positively charged nitrogen atom, which allows them to disrupt the cell membranes of microorganisms, leading to bacterial destruction.^34,35^ Their ability to effectively target a wide range of pathogens including bacteria, viruses, and fungi makes QASs particularly useful in the context of surface modification for implantable medical devices to prevent colonization and biofilm formation.^36,37^ This further helps in reducing the risk of subsequent infections and antibiotic resistance phenomena.^38,39^ The presence and length of a carbon atom chain in QAS exert a pivotal effect on the hydrophobicity of QAS-based compounds and then on their antibacterial activity. As reported, longer alkyl chains tend to interact more strongly with the lipid bilayer of bacterial membranes, and this interaction facilitates the insertion of the QAS molecules into the membrane of bacteria, leading to its disruption.^40^

Moreover, QASs have several desirable properties that make them suitable for surface modification applications^41^ including their stability, nonvolatility, and long-lasting effect, thus providing a sustained release of antimicrobial activity.^42^ They are also compatible with various types of materials, including titanium.^43^ Several studies report the physical adsorption of QASs onto the titanium surface.^44,45^ Titanium devices have been immersed in solutions containing the QASs^45^ or treated with mixtures containing QASs and biocompatible materials, such as polymers or hydroxyapatite,^46^ allowing the salts to adhere to the surface through electrostatic interactions. Even if physical adsorption has proved to offer significant antimicrobial benefits, these techniques may not provide the same level of durability as covalent bonding.^46,47^

In this study, to impart antibacterial adhesion properties to commercial titanium discs^21,48^ we covalently bonded QASs to titanium discs by using bifunctional silane coupling agents, able to bind the hydroxyl groups present on the titanium surface and with the reactive group of QAS molecules.^21,49^ The QASs chosen in this study were acryloyloxyalkyltriethylammonium bromides (AATEABs), and, in particular, acryloyloxyhexyltriethylammonium bromide (AHTEAB), acryloyloxynonyltriethylammonium bromide (ANTEAB), acryloyloxyundecyltriethylammonium bromide (AUTEAB), and acryloyloxydodecyltriethylammonium bromide (ADTEAB), which have been previously synthesized by us and proven to be good antibacterial agents.^50,51^ The quaternary ammonium salts (QASs) were stably bonded to the titanium disc surfaces using the linker (3-aminopropyl)triethoxysilane (APTES). The alkoxy groups of APTES reacted with the hydroxyl groups on the activated titanium discs, forming siloxane bonds, while the amino group of the linker was used for the conjugation reaction with the terminal double bond of the QASs.

In this work, we have demonstrated for the first time the effect of covalent functionalization on quaternary ammonium salt (QAS)-coated titanium discs featuring different carbon atom chain lengths, specifically examining their antimicrobial properties against resistant and sensitive strains of Gram-positive *Staphylococcus aureus*. From this study, it emerged that the chain length of the carbon substituents plays a significant role in enhancing the antimicrobial efficacy of the QAS-modified titanium surfaces. In particular, titanium discs functionalized with longer carbon chains exhibited a marked reduction in bacterial adherence and viability, particularly against antibiotic-resistant *Staphylococcus aureus* strains, compared to their shorter-chain counterparts. These results suggest that the physical and chemical interactions mediated by the varying hydrophobicities and surface energies of the QAS-modified titanium surfaces are pivotal in disrupting bacterial membrane integrity. Additionally, our results indicate a potential avenue for developing advanced antimicrobial surfaces in clinical settings, highlighting the need for optimized QAS formulations to combat infections caused by resistant bacterial strains.

## 2 Experimental Section

### 2.1 Materials and Methods

The titanium discs with a diameter of 25 mm and a thickness of 0.5 mm were purchased from Merck Life Science and feature chemical and mechanical properties that comply with the ASTM-B-265/ASME-SB-265 standard. 3-Aminopropyltriethoxysilane, hydrogen peroxide, sulfuric acid, and the solvents toluene (99.8%), ethanol (99.8%), and methanol (99.8%) were purchased from Merck Life Science and used without further purification. The ammonium salts AHTEAB, ANTEAB, AUTEAB, and ADTEAB were synthesized as previously reported.^50^ FT-IR analyses were recorded using a Fourier-transform infrared (FT-IR) Spectrum Two Spectrometer (PerkinElmer Inc., Waltham, MA, USA), by the ATR method in the range of 4000–500 cm^–1^. Scanning electron microscopy images (SEM) were performed with an FEI Quanta 450 FEG instrument (Thermo Fisher Scientific, Hillsboro, OR, USA) at room temperature and operating in high vacuum, at 20 kV, using an Everhart–Thornley detector (ETD). Energy-dispersive X-ray (EDX) analyses were carried out using the detector Octane Plus Silicon Drift (Ametek, Berwyn, PA, USA), equipped with a 30 mm^2^ super ultrathin window (SUTW). FeK mapping analyses were recorded with a dwell time of 200 μs and using an image resolution of 256 × 200 pixels. The mapping acquisition time was fixed at 60 min. The disc roughness was measured with a Surftest SJ-210 Series 178 roughness tester (Mitutoyo S.r.l., Milan, Italy). Specifically, the arithmetic mean height (Ra) [μm] was calculated by
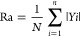
1where Ra refers to the arithmetic mean of the absolute values of the evaluation profile (*Yi*) with respect to the mean line. The measurement parameters were determined according to the JIS2001 roughness standard, five sampling lengths, shear lengths (λs = 2.5 μm, λc = 0.8 μm), and a stylus displacement speed of 0.5 mm/s. Six roughness profiles were recorded for each sample type, and an average profile was then calculated. Contact angle measurements were conducted using the static method with the tangent angle at the three-phase contact point of a sessile drop determined from high-resolution images of water droplets. Image analysis was performed by using Gimp software. Ten contact angle readings were taken for each sample, with droplets of 1 μL in volume. For each set of measurements, contact angles were recorded on both the left and right sides, with each side measured ten times. The final results presented are the averages of these measurements, derived from eqs 2 and 3:
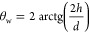
2
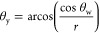
3where *d* is the diameter and *h* is the height of the drop (in mm), θ_w_ is the apparent Wenzel angle dependent on surface roughness, *r* represents the surface roughness (Ra), while θ_Y_ is Young’s contact angle of equilibrium on a perfectly smooth surface.

### 2.2 Functionalization of Titanium-Based Samples

**Figure 1:**
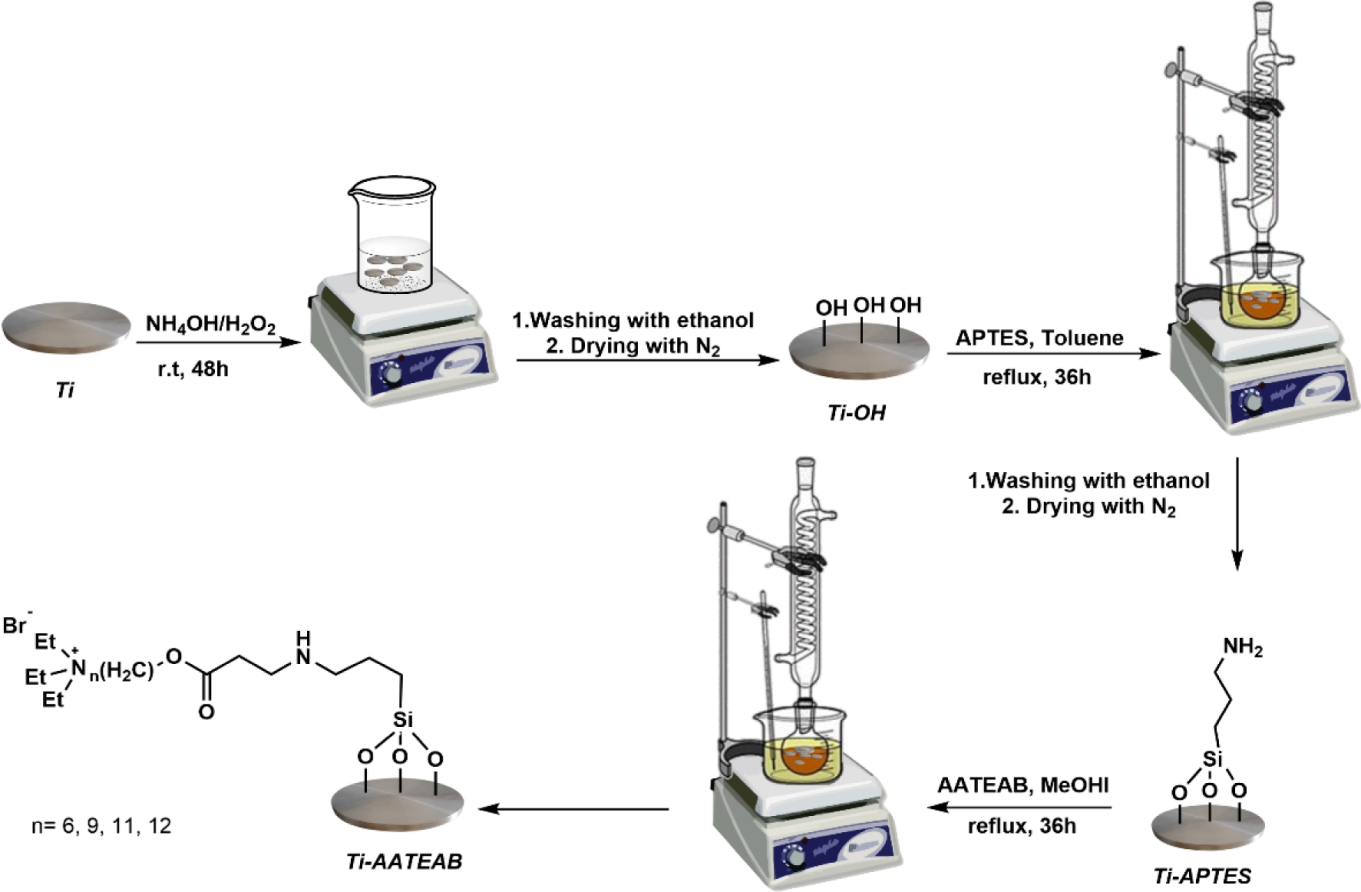
Schematic representation of the methodology employed for the preparation of titanium discs.

The titanium-based samples used in this study have been synthesized starting from commercial titanium discs by activation, silanization, and nucleophilic addition procedures, following the protocol reported below (Figure 1).

#### 2.2.1 Activation of Titanium Discs

The titanium discs were cleaned using ethanol and deionized water in an ultrasonic bath (10 min each). Then, the titanium disc surfaces were activated by soaking the discs for 48 h in a piranha base solution (100 mL), a 3:1 mixture of ammonium hydroxide (NH_4_OH), and hydrogen peroxide (H_2_O_2_). After activation, the discs were washed with ethanol and dried using nitrogen gas, thus obtaining the Ti–OH samples.

#### 2.2.2 Silanization of Activated Titanium Discs

Ti–OH samples were silanized using a 2.0 vol % solution of APTES in dry toluene, as previously reported by us.^21^ The samples were immersed in a glass flask equipped with a reflux condenser and heated at 70 °C for 36 h under nitrogen flux. Subsequently, the discs were washed with ethanol, dried with nitrogen, and labeled as Ti-APTES.

#### 2.2.3 Synthesis of AATEAB-Functionalized Titanium Discs

Ti-APTES samples were immersed in a flask equipped with a reflux condenser and treated with a solution of the AATEAB (AHTEAB, ANTEAB, AUTEAB, or ADTEAB, 0.28 mmol) in methanol (25 mL). The discs were heated at 80 °C for 36 h, then washed with ethanol, and dried under nitrogen flux. The so-synthesized samples were labeled Ti-AHTEAB, Ti-ANTEAB, Ti-AUTEAB, and Ti-ADTEAB, respectively.

#### 2.2.4 Cell Cultures

The human mammary fibroblast (HMF) cell line was obtained from Innoprot (Derio, Spain). HMF cells were routinely cultured in a humidified 5% CO_2_ incubator at 37 °C in Fibroblasts Medium supplemented with 2% fetal bovine serum (FBS), a mixture of antibiotics (streptomycin/penicillin), and Fibroblast Growth Supplement (FGS) (Innoprot, Derio, Spain).

#### 2.2.5 Evaluation of Cytotoxicity

To assess the cytotoxicity of HMF cells, the MTT colorimetric assay was used, based on the reduction of 3-(4,5-dimethylthiazol-2)-2,5-diphenyltetrazolium bromide. Briefly, functionalized and nonfunctionalized titanium discs were placed in 6-well microplates where cells were added in growth medium (1 × 10^5^ cells/well). After 24 h, the discs were placed in a second 6-well plate to check cell adhesion. MTT was added to both plates, and after incubation at 37 °C for 3 h, the enzyme activity of the viable cells was quantified by spectrophotometric measurement at 540 nm using a microplate reader (Tecan Italia, Milan).

#### 2.2.6 Evaluation of the Antibacterial and Antibiofilm Activities of Functionalized Titanium Discs

To evaluate the antibacterial properties of functionalized titanium discs, American Type Culture Collection (ATCC) strains of methicillin-sensitive (MSSA, Newman D2C [NCTC 10833]) and methicillin-resistant (MRSA, TCH1516 [USA300-HOU-MR]) *Staphylococcus aureus* were used. Bacteria were first cultured in Brain-Heart Infusion (BHI) broth (Merck KGaA, Darmstadt, Germany) at 37 °C for 24 h. From primary cultures, a 0.5 McFarland solution of both strains was obtained, and a plate count method on Petri dishes, including in the middle the titanium disc, was performed. Specifically, on 35 mm Petri dishes, after inoculation of 10 μL of the 0.5 bacterial suspension on the surface of the disc, 1 mL of melted Plate Count Agar (PCA) (Merck KGaA, Darmstadt, Germany) was added in order to obtain an extremely thin layer of agar above the disc. After solidification, Petri dishes were incubated at 37 °C for 24 h. After the incubation period, colonies were imaged with a digital camera and quantified using the free program ImageJ (imagej.nih.gov/ij/index.html).^52^ For the antibiofilm activity, nonfunctionalized and functionalized discs were put in contact with a 0.01 McFarland bacterial suspension of both strains and incubated at 37 °C for 48 h. Then, after being washed three times with sterile PBS in order to remove all the suspended bacteria, discs were sonicated (Branson 3210 Ultrasonic Cleaner) for 20 min in 5 mL of sterile PBS to detach the eventually formed biofilm from the surface of the disc. From the collected suspension, 10-fold serial dilutions were obtained and plated on PCA for the count. Plates were then incubated at 37 °C for 24 h, and colonies were quantified using the free program ImageJ. At the same time, from the collected suspensions, we set up slides to obtain confocal microscopy images of the bacterial biofilm. Specifically, bacteria were fixed by heat and stained with propidium iodide (20 μg mL^–1^) (Sigma-Aldrich, Italy), a nucleic acids’ intercalating and fluorescent agent. The slides were incubated in the dark at 30 °C for 5 min to enable the fluorescent labeling of the bacteria. Analyses were carried out through CLSM using a TCS SP2 microscope, equipped with an Ar/Kr laser (Leica Microsystems Heidelberg, Mannheim, Germany), and coupled to a microscope (Leica DMIRB). The fluorescence excitation maximum was 535 nm, and the emission maximum was 617 nm. All of the experiments were performed in triplicate.

#### 2.2.7 Statistical Analyses

Statistical analyses were executed with Prism 4.0 software (GraphPad, San Diego, CA, USA). Stratified data were statistically calculated using one-way ANOVA and *t* test. The significance analysis was estimated at the *p* < 0.05 level.

## 3 Results and Discussion

### 3.1 Titanium Discs’ Activation

**Figure 2:**
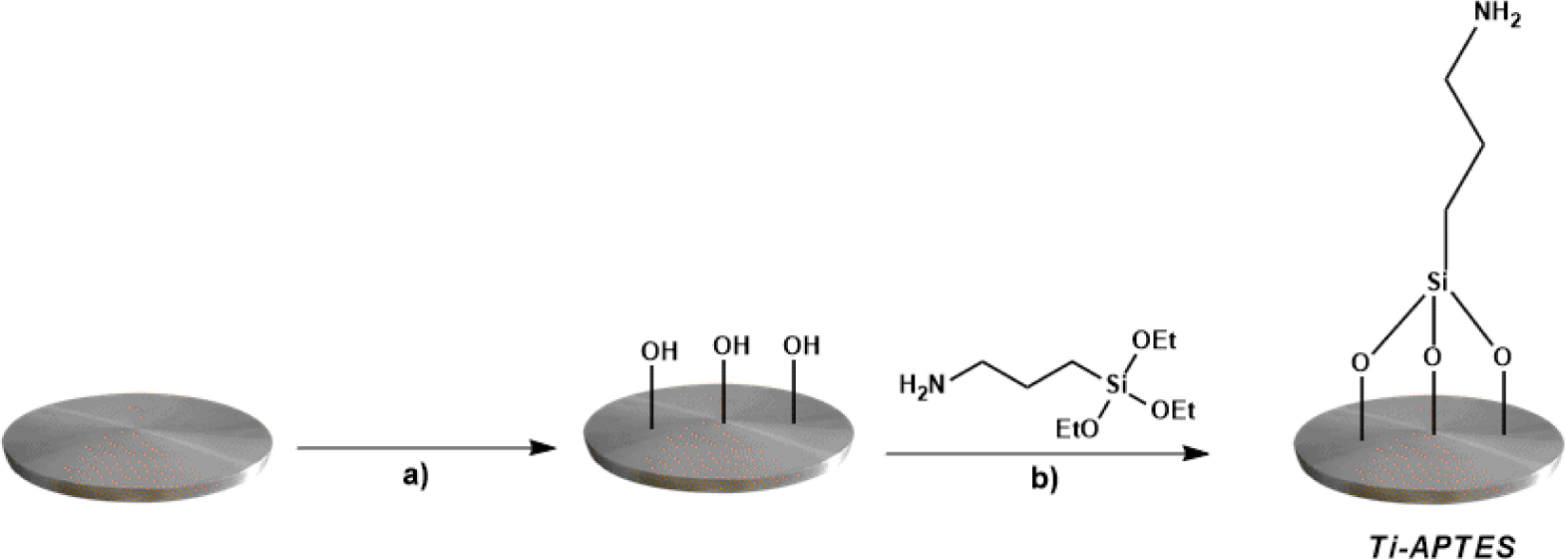
Activation and silanization of titanium disc surfaces: (a) NH_4_OH/H_2_O_2_, 48 h rt; (b) (3-aminopropyl)triethoxysilane, toluene, 36 h, reflux.

The potential for creating intrinsically antibacterial titanium metal substrates relies on the silanization of the titanium surface to anchor the selected bioactive molecules. This method takes advantage of the spontaneous formation of strong siloxane bonds from the reaction between the alkoxy groups of APTES and the hydroxyl groups of titanium, while the nucleophilic amino group facilitates further bonding with ammonium salts (Figure 2).

**Figure 3:**
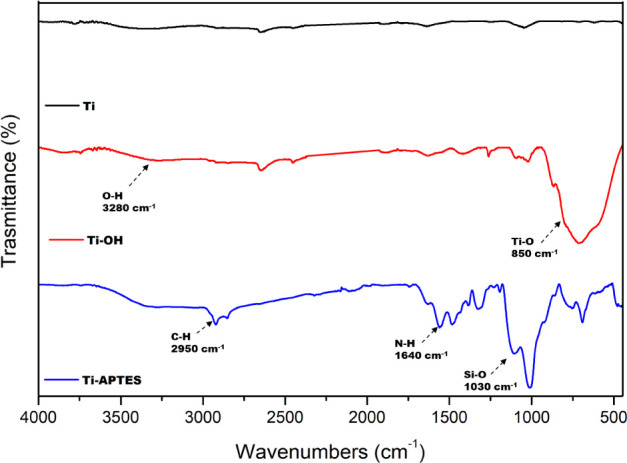
FT-IR spectra of bare titanium sample Ti, oxidized sample Ti–OH, and silanized sample Ti-APTES.

To achieve optimal silanization, the surface of the discs was activated by basic treatment using a mixture of NH_4_OH/H_2_O_2_ for 48 h. This oxidative treatment enhanced the number of hydroxyl groups available, as demonstrated by the FT-IR spectra (Figure 3). In comparison to the untreated sample (Ti), the Ti–OH sample exhibits a peak at 3280 cm^–1^, corresponding to the vibration of the O–H groups, and a band at 850 cm^–1^, typically associated with Ti–O bonds. The subsequent treatment of Ti–OH with the silane APTES, performed in anhydrous toluene by heating the discs at 70 °C for 36 h under an inert atmosphere, resulted in the silanized Ti-APTES discs. The effective functionalization was also demonstrated by the same technique. Indeed, the FT-IR spectra of these samples showed the representative peaks at 1030 cm^–1^, ascribable to the Si–O bond and the C–H aliphatic stretching of the propyl group at 2930–2950 cm^–1^, so indicating that the silane moieties were covalently bound to the surface of the substrate. Additionally, these samples showed a peak at 3340 cm^–1^, attributed to the symmetric and asymmetric vibrations of the −NH group, along with peaks at 1530 and 1640 cm^–1^, corresponding to the bending of the N–H group of Ti-APTES. Compared to the Ti–OH samples, the Ti-APTES sample exhibited a weaker, broader peak at 3350 cm^–1^, as a result of the silanization process.

**Figure 4:**
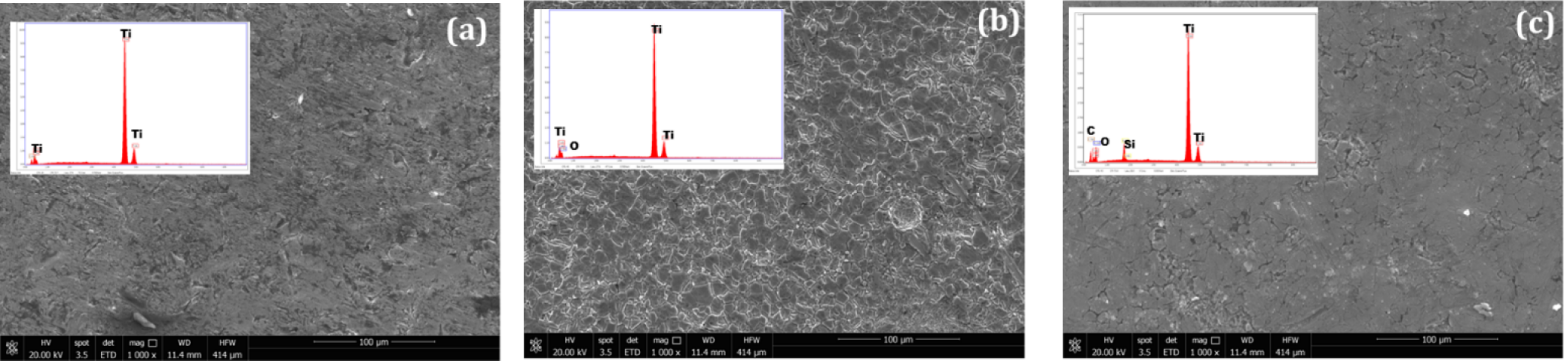
Representative scanning electron microscopy/energy-dispersive X-ray (SEM/EDX) analyses of (a) Ti, (b) Ti–OH, and (c) Ti-APTES samples.

**Figure 5:**
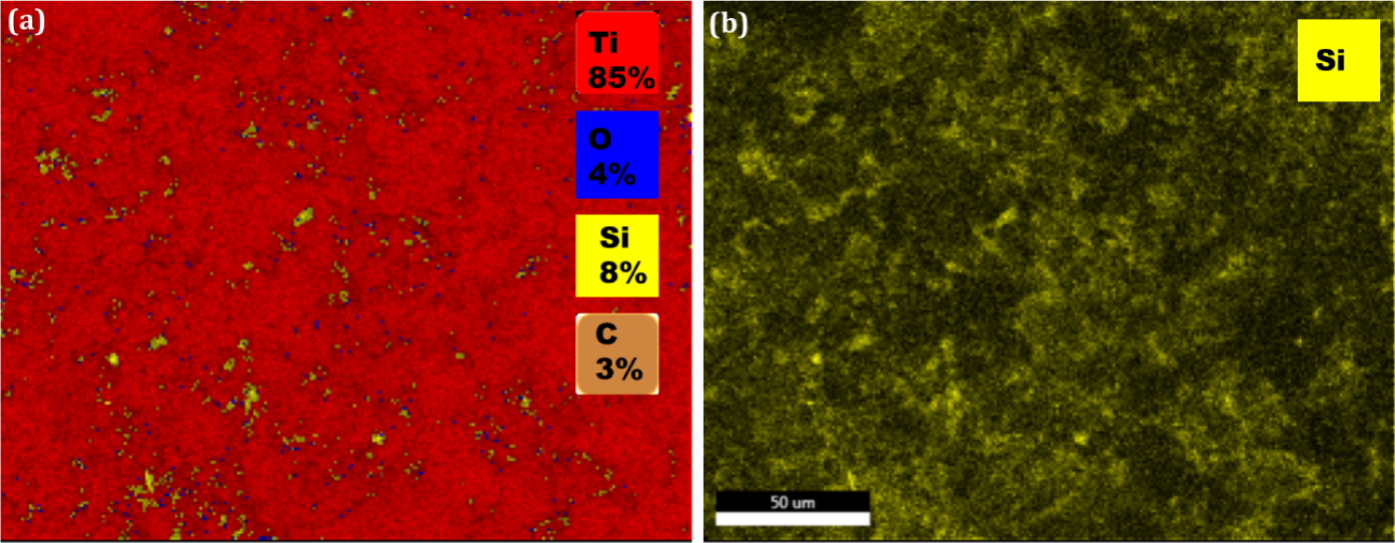
(a) EDX mapping analysis performed on Ti-APTES; (b) presence of silicon atoms (yellow spots) on Ti-APTES.

Both titanium surface activation by basic treatment and the effective functionalization by the silanization process were also proved by field emission gun scanning electron microscopy (FEG-SEM) (Figure 4). As also reported in similar studies,^53,54^ the chemical treatment of titanium surfaces with basic mixtures, in addition to improving the biointegration properties, incisively modified the surface of the discs by changing the initial morphology of the substrates. The existence of an amorphous alkaline titanate layer was verified by EDX spectra, which indicated the presence of oxygen peaks for the oxidized sample. Elemental analysis via EDX indicated an atomic mean percent oxygen content of 4%. The following silanization procedure revealed the presence of homogeneous silicon atoms regularly covering the surfaces of the discs, with an average silicon atom content of 8% for the Ti-APTES sample. The mapping analyses for the silanized Ti-APTES sample revealed the presence of homogeneously distributed silicon atoms (yellow dots) covering the surfaces of the substrates (Figure 5).

### 3.2 Titanium Discs’ Functionalization

**Figure 6:**
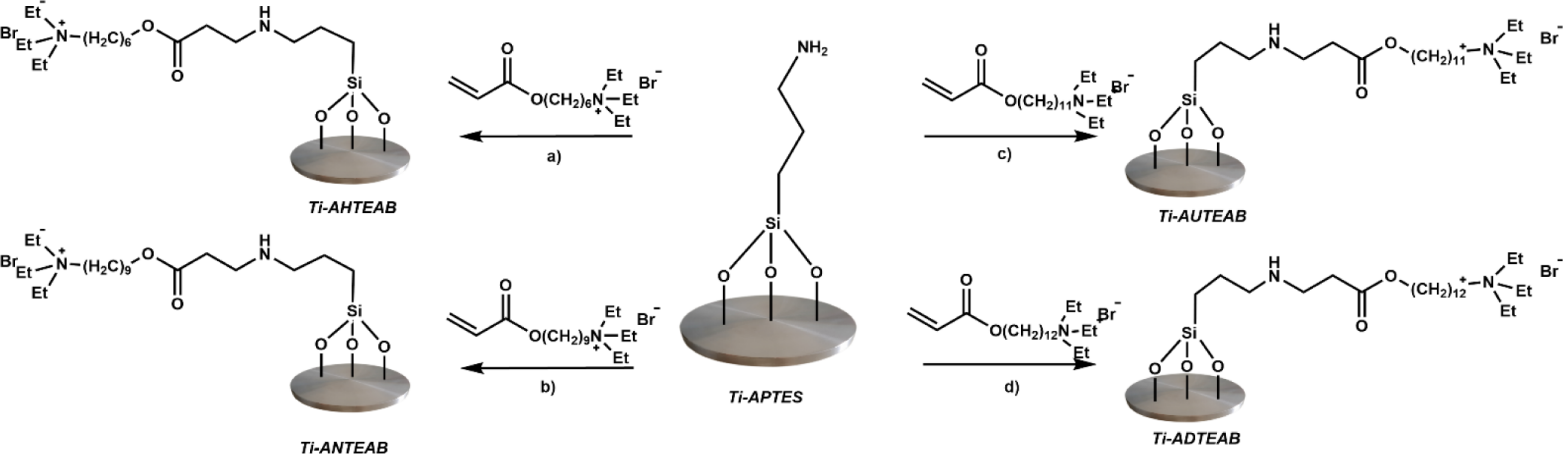
Schematic diagram illustrating the synthetic routes for titanium surface functionalization: (a) AHTEAB, methanol, 36 h, reflux; (b) ANTEAB, methanol, 36 h, reflux; (c) AUTEAB, methanol, 36 h, reflux; and (d) ADTEAB, methanol, 36 h, reflux.

The Ti-APTES samples thus obtained were further functionalized with the bioactive QAS molecules (AATEABs) to impart intrinsic antibacterial activity to the titanium metal surfaces. The QASs used, namely, AHTEAB, ANTEAB, AUTEAB, and ADTEAB, were covalently anchored to the Ti-APTES sample via conjugate addition of the free amino group to the terminal double bond of the QASs (Figure 6).

**Figure 7:**
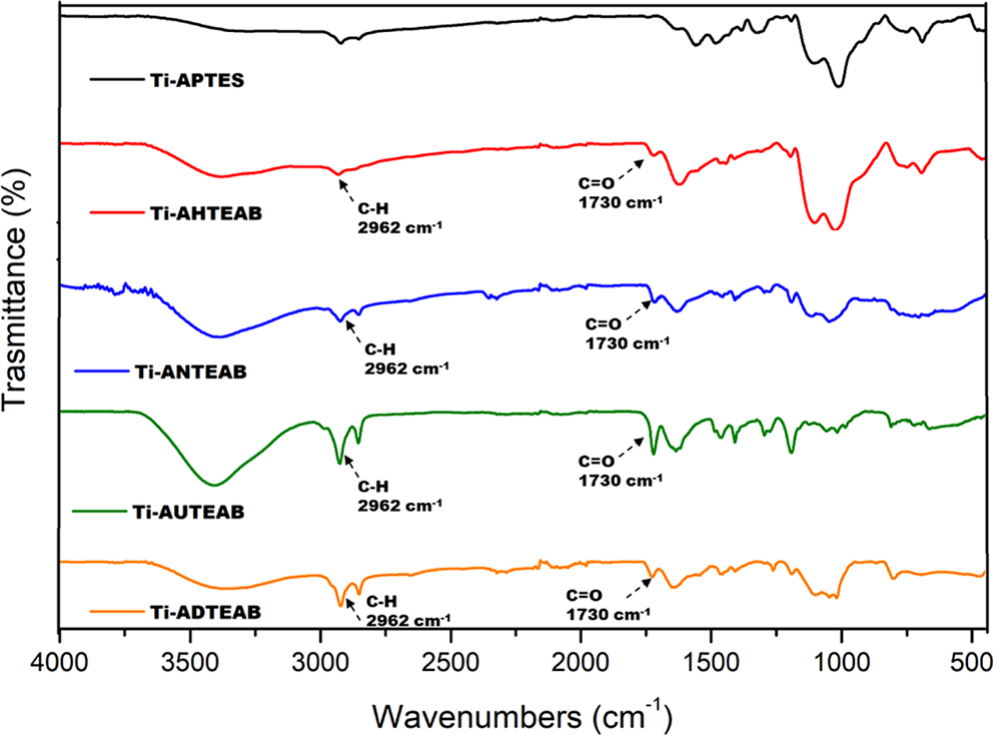
FT-IR spectra of Ti-AHTEAB, Ti-ANTEAB, Ti-AUTEAB, and Ti-ADTEAB samples, compared with the Ti-APTES sample.

The successful functionalization reaction was confirmed by FT-IR analysis (Figure 7). The functionalized samples endowed with quaternary ammonium functionality all display a broad band at 3000–2800 cm^–1^ attributable to the stretching of the N–H bond of the amine salt introduced and a more pronounced peak, compared to the Ti-APTES sample, at 2940–2930 cm^–1^, due to C–H bending of the alkane functionalities (Figure 7). All samples also show the presence of a peak at 1730 cm^–1^, due to the C=O binding of the ester moieties. Furthermore, these samples exhibit peaks at 1130 cm^–1^ due to the stretching of the C–O groups.

**Figure 8:**
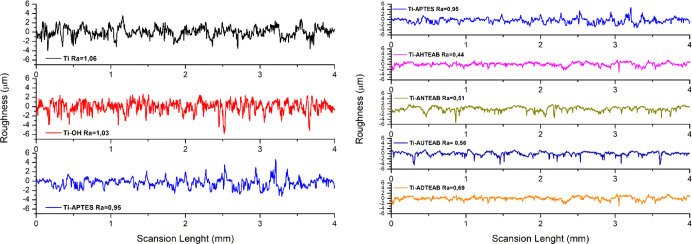
Surface roughness profiles of Ti, Ti-AHTEAB, Ti-ANTEAB, Ti-AUTEAB, and Ti-ADTEAB samples.

The profiles and surface roughness parameters of these materials (Ra) are presented in Figure 7. The functionalization process significantly alters the surface roughness, which in turn affects the interaction of the metallic substrate with the surrounding biological systems. These values align well with the results obtained from the SEM analysis (Figure 4). In particular, activation by treatment with the ammonium hydroxide/hydrogen peroxide mixture led to an increase in surface roughness (Ra value) from 1.06 μm in the unmodified Ti sample to 1.80 μm in the activated Ti–OH sample. The silanization procedure resulted in a decrease in the Ra value to 0.95 μm for Ti-APTES, further confirming the presence of homogeneously distributed silicon fractions. The functionalization procedure should lead to a decrease in surface roughness compared to an unmodified matrix; indeed, the Ti-AHTEAB, Ti-ANTEAB, Ti-AUTEAB, and Ti-ADTEAB samples exhibited reduced roughness with Ra values of 0.44, 0.51, 0.56, and 0.69 μm, respectively, confirming the effectiveness of the functionalization procedures (Figure 8).

**Figure 9:**
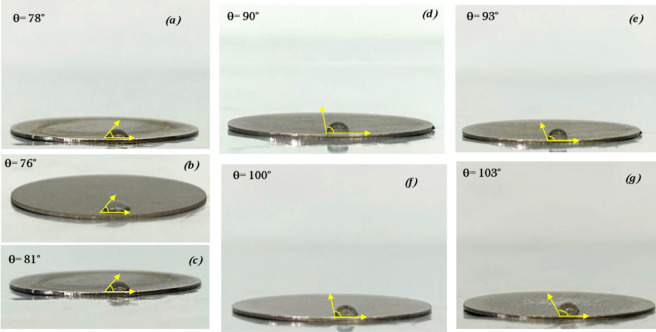
Comparison of contact angles θ_w_ and θ_y_ of (a) Ti, (b) Ti–OH, (c) Ti-APTES, (d) Ti-AHTEAB, (e) Ti-ANTEAB, (f) Ti-AUTEAB, and (g) Ti-ADTEAB samples.

To gain a clearer understanding of how the chemical structure of the coating influences its properties, wettability and surface roughness should be measured at the same point on the sample. The results can then be analyzed using eq 3 to distinguish the effects of roughness from those of wettability. Figure 9 shows the contact angle values (θ) provided by Wenzel’s equation and Young’s equation for functionalized titanium discs. Hydrophobicity stands out as a crucial surface characteristic, significantly influencing cell adhesion. This effect can be explained by considering that hydrophobic surfaces not only repel water but also inherently display structures that restrict bacterial contact, thereby also exhibiting antibacterial adhesion properties.^55−57^ As shown in Figure 9, the Wenzel contact angles of all tested samples increase from 90° to 103°, indicating that the surfaces have hydrophobic properties. The increase in hydrophobicity observed for the samples can be rationalized by the content of alkyl chains that increase in length as the hydrophobic properties increase.

**Figure 10:**
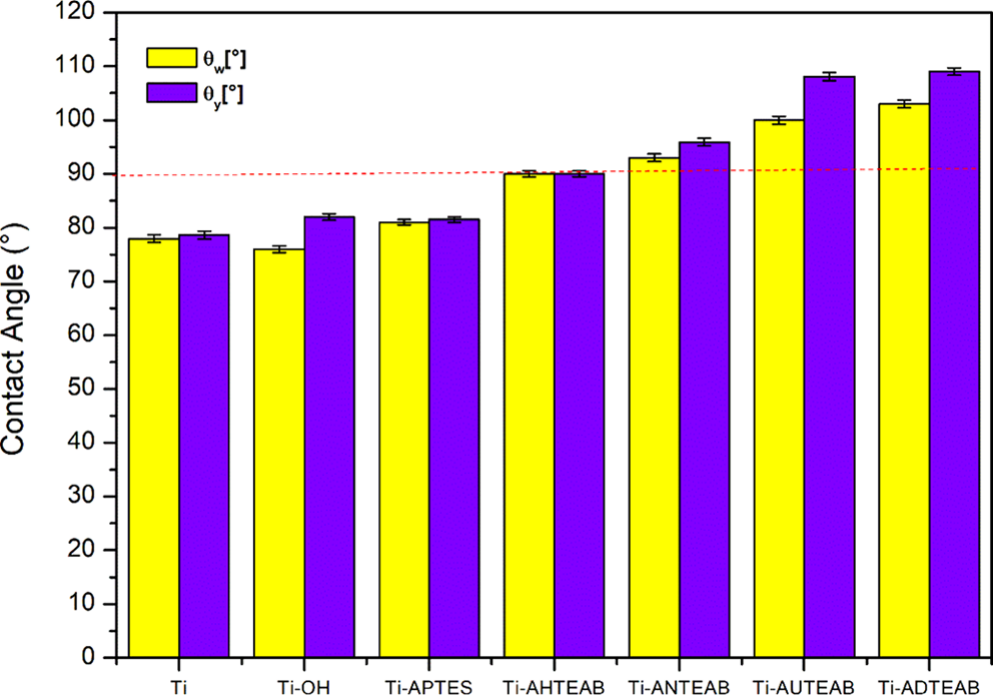
Comparison of contact angles θ_w_ and θ_y_ of the titanium disc.

As shown in Figure 9 and Table 1, all functionalized titanium discs exhibit significantly higher contact angles (and thus reduced wettability) compared with the untreated Ti sample, which has a contact angle of less than 90°. The apparent static Wenzel contact angle (θ_w_) is influenced by surface morphology. Since every surface is heterogeneous, the contact angle θ_w_ measured when the liquid fully contacts the substrate differs from the ideal Young’s contact angle (θ_y_). Hydrophilic coatings have a contact angle (θ) of less than 90°, while hydrophobic coatings have θ > 90°. As a result, the Ti sample’s hydrophilic characteristics change to hydrophobic after the silanization and functionalization processes. The value of the ideal contact angle of each sample, represented by Young’s value (θ_y_), is always higher than Wenzel’s value (θ_w_); see Figure 10 and Table 1. This is because it refers to an ideal “perfectly smooth” surface. As can be seen, as the length of the long hydrocarbon chain of the quaternary ammonium salts increases, there is a significant increase in the contact angle of the treated substrates. Therefore, from this study, it emerges that, rather than the presence of cationic ammonium salts, it is the length of the hydrocarbon chain that influences the hydrophobic behavior of the coating samples.

**Table 1:** Roughness Value of Ra and Comparison of the Contact Angles of Wenzel (θ_w_) and Young (θ_y_) of the Titanium Disc

Sample	Ra (μm)	θ_w_ (deg)	θ_y_ (°/μm)
Ti	1.06 ± 0.042	77.95 ± 0.75	78.64 ± 0.75
Ti–OH	1.03 ± 0.046	76.00 ± 0.63	76.42 ± 0.63
Ti-APTES	0.95 ± 0.04	81.00 ± 0.49	80.52 ± 0.49
Ti-AHTEAB	0.44 ± 0.011	90.01 ± 0.54	90.01 ± 0.54
Ti-ANTEAB	0.51 ± 0.010	93.05 ± 0.70	95.89 ± 0.70
Ti-AUTEAB	0.56 ± 0.070	100.12 ± 0.74	108.06 ± 0.74
Ti-ADTEAB	0.69 ± 0.061	103.06 ± 0.68	109.03 ± 0.68

### 3.3 Cytotoxicity, Antibacterial, and Antibiofilm Activity of Functionalized Titanium Discs

**Figure 11:**
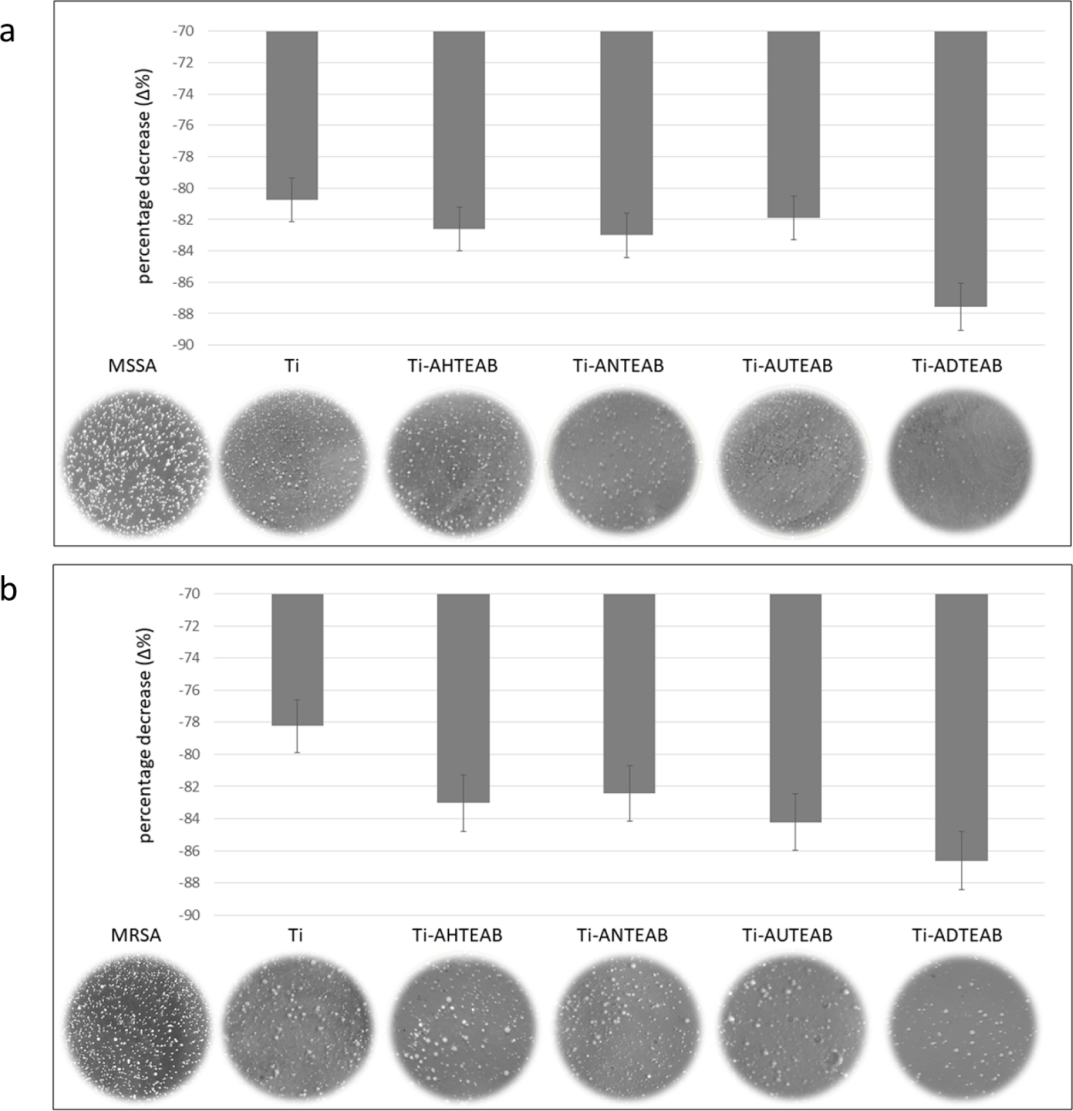
Antibacterial activity of nonfunctionalized and functionalized titanium discs: MSSA (a) and MRSA (b).

The cytotoxicity of functionalized titanium discs evaluated by the MTT assay showed a moderate increase of mortality proportional to the salt chain length. Specifically, the percentage of mortality of both the cells adherent to and around the disc, compared to untreated cells, was 11.5 ± 1.2 for Ti-AHTEAB, 28.7 ± 2.1 for Ti-ANTEAB, 29.8 ± 2.6 for Ti-AUTEAB, and 29.9 ± 3.1 for Ti-ADTEAB. To evaluate the suitability of the proposed functionalization strategy for the development of intrinsic antimicrobial titanium devices, biological tests were performed against antibiotic-susceptible and resistant *S. aureus* (MSSA and MRSA, respectively) strains, an important human bacterial pathogen causing a wide variety of clinical manifestations.^58^ The bacterial growth inhibition activity, compared to the growth of the used standard strains, showed significant antibacterial activity for all samples, with some differences among them (Figure 11a,b).

Specifically, referring to nonfunctionalized titanium discs, the antibacterial activity was practically superimposable for both strains (−80.7 and −78.2% for MSSA and MRSA, respectively), but lower than that shown by functionalized discs. In particular, for the latter, the antibacterial activity was correlated with the length of the QAS chain, with longer-chain compounds being more active than short-chain ones. In fact, among functionalized titanium discs, the longest chain compound (Ti-ADTEAB) showed the highest percentage decrease for both strains (−87.5 vs 86.6% for MSSA and MRSA, respectively). This effect of increased activity related to chain length was clearly visible in our experimental conditions, and it was found for both of the strains used.

**Figure 12:**
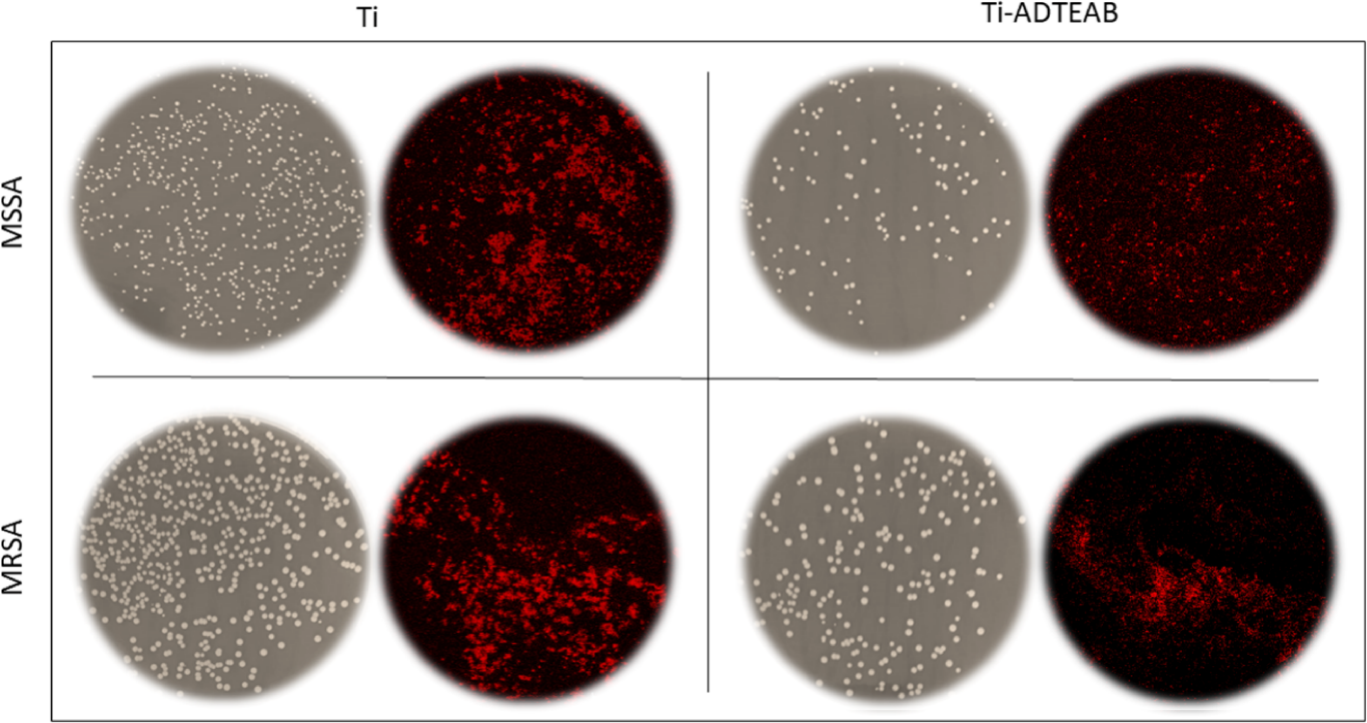
Antibiofilm activity of nonfunctionalized and Ti-ADTEAB discs in MSSA and MRSA as PCA plates count and confocal microscopy images.

Concerning antibiofilm activity, the obtained results were very similar, reporting a number of colonies higher in the sonicated solution collected from nonfunctionalized discs compared to that collected from functionalized ones. These results were observed for both strains and were particularly accentuated when comparing the nonfunctionalized disc with Ti-ADTEAB. Specifically, the number of colonies was equal to 1,950 × 10^6^ CFU/mL (nonfunctionalized disc) vs 437 × 10^6^ CFU/mL (Ti-ADTEAB) for the sensitive strain and 1,995 × 10^6^ CFU/mL (nonfunctionalized disc) vs 845 × 10^6^ CFU/mL (Ti-ADTEAB) for the resistant one with a percentage decrease compared to the nonfunctionalized disc of −77.5% for the sensitive strain and −57.6% for the resistant one. These differences were confirmed by confocal microscopy images (Figure 12).

Overall, from both the performed typologies of experiments, we observed good antibacterial and antibiofilm activity of functionalized titanium discs compared to nonfunctionalized ones. However, it has to be stressed that some differences were found between the sensitive and resistant strains. Indeed, while for the antibacterial activity we obtained superimposable results against the two different typologies of used strains, in the experiments of antibiofilm activity, the latter was less marked for the resistant strain that resulted more capable of producing biofilm on the functionalized disc than the sensitive strain, in line with some previous literature data.^59,60^ Despite this, we can say that functionalized titanium discs showed an important antibiofilm activity also for the resistant strain, and this is a very interesting finding due to the fact that many cases of implant infections characterized by severe clinical outcomes are caused, nowadays, by antibiotic-resistant strains. Therefore, our antimicrobial and antibiofilm effects also toward antibiotic-resistant bacteria represent an important goal in reducing the risks of titanium implant infections. This finding allowed us to make an important practical consideration. Despite the intrinsic antibacterial activity shown by some materials used in the manufacturing of medical devices, it can be essential to potentiate this clinically relevant property with some active compounds, and the QASs used in this study could represent a valid long-term solution.

## 4 Conclusion

In summary, the functionalization of titanium surfaces with quaternary ammonium salts (QASs), acryloyloxyalkyltriethylammonium bromides (AATEABs) in particular, has been shown to effectively provide antibacterial properties to implantable medical devices with acceptable cytotoxicity. Whether through chemical immobilization, physical adsorption, or incorporation into coatings, QASs offer potent antimicrobial activity and can help reduce the risk of bacterial colonization and infection. Here, we have reported the surface modification of titanium discs with AATEABs containing 6–12 carbon atom alkyl chains using APTES as a cross-linking agent between the hydroxyl groups of activated titanium discs and the terminal double bond present in the AATEABs. The effect of chain length was investigated here for the first time to address the antimicrobial properties of resistant and sensitive Gram-positive *Staphylococcus aureus* strains, demonstrating the best antibacterial performance for the sample containing the longest carbon chain tested (C-12), despite the higher mortality rate, which is however contained. These modifications can prevent bacterial attachment and biofilm formation for antibiotic-sensitive and resistant bacteria, leading to improved patient outcomes and increased implant longevity.
